# Short-term impacts of forest clear-cut on soil structure and consequences for organic matter composition and nutrient speciation: A case study

**DOI:** 10.1371/journal.pone.0220476

**Published:** 2019-08-01

**Authors:** Nina Siebers, Jens Kruse

**Affiliations:** 1 Agrosphere Institute, Institute of Bio- and Geosciences (IBG-3), Forschungszentrum Jülich, Jülich, Germany; 2 Institute of Crop Science and Resource Conservation (INRES), Soil Science and Soil Ecology, University of Bonn, Nussallee, Bonn, Germany; RMIT University, AUSTRALIA

## Abstract

Clear-cuts of forests severely affect soil structure and thus soil organic matter (SOM) and nutrient cycling dynamics therein, though with yet unknown consequences for SOM composition as well as phosphorus (P) and sulfur (S) chemical form within the soil microaggregate size fraction. To determine the effects of conventional clear-cutting on soil chemistry in a Cambisol of the Wüstebach Forest (northwestern Germany), we sampled the mineral A- and B-horizons prior to clear-cut as well as 10 and 24 month thereafter. We measured the SOM composition of soil microaggregates using pyrolysis field ionization mass spectrometry (Py-FIMS), as well as P and S chemical form and speciation using wet-chemical extractions and X-ray absorption near edge structure (XANES) spectroscopy. We found that clear-cut led to an increase of the microaggregate size fraction up to 6% due to break-down of macroaggregates and initially significantly increased total elemental concentrations (C, N, P, S) due to the introduction of slash-residues. The SOM of slash-residues consisted to a substantial amount of sterols and was generally found to be of low thermal stability and probably did not contribute to aggregate stability. Deterioration of the aggregate structure probably led to an exposure of originally inaccessible sites within aggregates to the attack by soil microorganisms and thus to an increased P and S turnover as reflected in a significantly reduction of available P proportions (4 to 7%) and a reduction of the most reduced S forms (5%). A probable increased microbial activity and contribution to SOM after clear-cut is also reflected in the significantly increasing hexose:pentose ratio by 0.25 between 10 and 24 month after clear-cut, significantly increasing the general thermal stability of SOM in the microaggregate size fraction and believed to contribute to aggregate stability. This indicated that a first deterioration of the aggregate structure after clear-cut might in the long-term be remediated with time.

## 1 Introduction

As outlined in the United Nations Millenium Assessment, our ecosystems are increasingly prone to non-linear changes [1]. Such developments are hard to predict, its process understanding thus calling for long-term data sets, as currently collected in terrestrial observatories around the globe. One of these long-term terrestrial observatories is TERENO, which spans an Earth observation network across Germany (www.TERENO.net). These observatories supply important data for responding to the impact of long-term climate and land-use change on the ecosystems [2]. The TERENO Wüstebach site is facing such a recent management change, namely a clear-cutting. In general, clear-cuttings of forests have severe effects on the soil structure, carbon (C) storage, and nutrient dynamics [3–7].

Beside single primary particles, most soils are composed of so-called aggregates, secondary structural units which themself are hierarchically composed of smaller units separated by persistent planes of weakness or glued together by a range of different bonding agents [8]. Within the soil aggregates system, there are generally two kinds of operational defined soil aggregate sizes, namely soil microaggregates <250 μm and soil macroaggregates >250 μm [8]. Microaggregates are strongly linked with major biochemical processes controlling the turnover of elements in the soil [9,10]. Soil macroaggregates (>250 μm) finally consist of such microaggregates [8], stabilized by temporary binding agents, e.g., roots, extracellular polymeric substances (EPS), and EPS born biomolecules [11]. These soil macroaggregates are easily susceptible to disaggregation as a result of, e.g., land-use changes, whereas soil microaggregates reveal the highest stability and persistence fraction in the hierarchy of soil aggregates [12]. Microaggregates are less vulnerable to erosion than macroaggregates, internal abrasion, and soil organic matter (SOM) and nutrients bound to soil microaggregates may have relatively long turnover rates [9,10].

The concentration and composition of SOM is beside others (e.g., pH, nutrient availability, and microbial biomass) widely accepted as one indicator of forest soil quality (as reviewed by Schoenholtz et al. [13]). It is also one of the key components essential for aggregate formation and stabilization [9], influences soil porosity[14]. Soil organic matter also serves as binding partner as well as reservoir of essential nutrient elements such as nitrogen (N), phosphorus (P), and sulfur (S) and thus plays a pivotal role in nutrient release and availability [13]. Losses in SOM and nutrients due to changes of the aggregate structure as a result of land-use changes are well documented. For instance, the clear-cutting of a forest site is one of the most drastic changes in land-use leading to changes in physical soil properties such as moisture content or the aggregate structure, which facilitates release of soil particles and finally increasing risks of nutrient losses, [3–6,15–20]. In literature losses of up to 27% of total N [21], 32% of total P [19], or 63% of total S [22] as a result of clear-cutting are documented. These decreased levels of nutrients are first of all due to loss of biomass input into the soil and secondly due to changes in the soil structure [3,5,6,15,16], including the disintegration of soil macroaggregates into microaggregates [23]. While SOM is indispensable for a healthy and functioning soil that stores nutrients efficiently and provides them to plants when needed, at present only limited information is available on the mechanisms of SOM, P, and S alterations in the microaggregate size fraction of forest soils with recent change in land-use, particularly regarding those mechanisms that are linked to soil structure dynamics.

A well-established approach for the non-targeted molecular-chemical characterization of SOM is pyrolysis-field ionization mass spectrometry (Py-FIMS) [24]. The temperature resolved mass spectra obtained provide molecular information about abundance and origin of building blocks of SOM-thermograms obtained simultaneously reveal the strength of the chemical bonding within organic molecules, or between the SOM and mineral particles [25]. The amount and also the molecular-chemical characteristics of SOM are crucial for the aggregate structure stability and thus for the availability of nutrients stored therein. We hypothesize that clear-cutting deteriorates soil macroaggregate structure thereby exposing previously occluded and thus protected SOM being more susceptible to degradation. This, in turn, will influence the availability of key nutrients like P and S.

The nutrient availability is to a great extent influenced by its chemical form in soil. The chemical solubility can be assessed by using wet-chemical approaches such as single or sequential fractionations. Using the sequential fractionation approach by using subsequently stronger extraction solutions and thus obtaining P-pools of different availability as proposed by Hedley et al. [26] it is also possible not only to determine P but also to determine soil S within individual extracts, which can be assigned to operationally defined fractions of different availability [27,28]. These wet-chemical sequential extraction procedures are suitable for a large sample set as they are easy to apply and inexpensive; however, they only yield operationally defined fractions, limiting the assignment of a fraction to a specific chemical form [29,30]. Therefore, these methods are applicable to obtain an overview about P and S availabilities in environmental samples, but to reveal changes in the P and S binding forms other complementary techniques have to be applied. The X-ray absorption near edge structure (XANES) spectroscopy provides an element-specific and non-invasive direct elemental speciation in the solid phase [31]. In summary, the combination of wet-chemical fractionation approaches with state-of-the-art spectroscopic techniques enables a more detailed insight into the changes of the P and S speciation and thereby availability on soil microaggregates as influenced by clear-cutting.

In this case study, we took top- as well as subsoil samples of a forest soil before and recently after (10 and 24 month) a clear-cut and isolated the soil microaggregate size fraction, which was further analyzed regarding its SOM composition as well as P and S availability and speciation. With this we aimed at (i) estimating the changes in the aggregate structure in the soil and the composition of SOM in the soil microaggregate size fraction of top- and subsoil samples after clear-cutting, and (ii) relate the changes in aggregate structure and SOM composition to P and S availability.

## 2 Materials and methods

### 2.1 Site characteristics and experimental design

Soil samples were obtained from the study forest Wüstebach (50°30’15”N, 6°18’15”E) which is a part of the long-term terrestrial monitoring program TERENO (www.TERENO.net) that incorporates the complete Wüstebach catchment (38.5 ha). As the Wüstebach catchment belongs to the Eifel National Park, the Eifel National Park as the competent authority had approved the soil sampling at the Wüstebach test site. The mean annual temperature for this site is 7°C and the mean annual precipitation is 1220 mm [2]. The soil is a Cambisol (silt loam) with a soil texture of 21.3% clay, 60.6% silt, and 18.1% sand [10]. Forest development activities in the Eifel National Park encourage the regeneration of a near-natural beech forest, particularly in the spruce-monoculture-dominated south. On behalf of the national park forest management, spruce trees were therefore systematically removed in an area of 8.6 ha of the Wüstebach study site during late summer/early autumn of 2013, which equals about 22.3% of the total Wüstebach catchment. Clear-cutting was done with light machines and working lanes required for machines were covered with wood to minimize compaction. Furthermore, locations of working lanes were chosen to prevent physically disturbance of monitor/sampling plots. The average tree density was 370 trees per ha, with a dry aboveground forest biomass of 30 kg m^-2^ [32]. The prevailing vegetation type is norway spruce (*Picea abies*) and sitka spruce (*Picea sitchensis*) that was originally established for timber production after 1946 [33]. The deforestation measure included the removal of stems only, while roots were left in the ground so that the soil was disturbed at a minimum. The clear-cutting area was focused on the wettest part of the catchment near the main Wüstebach stream. It is expected that the biogeochemical processes and functioning of the forest ecosystem are significantly influence [2].

The experimental design is based on sampling soil from the forest site before (here referred to as native) and after a clear-cut event. Samples were taken during summer time (June to August) in 2013, 2014, and 2015. For each sampling date samples were taken prior to the deforestation measure at the native site (n = 6) and inside the future clear-felling area (n = 9). A different number of sample spots was selected for the native and the clear-felling site, as the sampling locations were assigned to the existing points of a wireless soil sensor network that were set up to support geostatistical analysis (SoilNet, 143 locations; [33]). In order to ensure comparability we have selected points within the network for this study that were all located at the same elevation, all on Cambisol (World Reference Base for Soil Resources) [34], and all exhibiting comparable soil texture. Thereby, six suitable sites could be identified for the native, whereas on the clear-felling site nine sites could be selected. Samples were taken as described in Siebers et al. [10]. The L/Of- and Oh-horizon was removed and the mineral horizons below were sampled at depth of 0 to 30 cm and 30 to 60 cm incorporating the two upper mineral horizons (A- and B-horizons, [34] subsequently referred to as top- (A-horizon) and subsoil (B-horizon) using a mineral soil corer as described in Wu et al. [35]. The cores were separated into the two horizons with a thickness of the A horizon of about 10 cm [36]. Samples taken in 2013 on the clear-felling area were taken shortly before the clear-cut. As the sampling was destructive, the sampling place was slightly shifted from the initial sampling for every year within an area of 40 x 40 cm [35].

### 2.2 Sample preparation, characterization, and elemental analyses

Samples were immediately dried after sampling, and if that was not possible frozen until further sample treatment. Dried samples were subsequently dry-sieved over aperture sieves to <2 mm and then <250 μm to obtain the microaggregate size fraction of the top- and subsoil samples. Sieving was done as described in Siebers et al.[37]. We are aware that sieving always holds the risk of abrasion and mechanical break-down of aggregates; in order to minimize this risk we gently placed the dried sample on top of the sieve and slowly shaken it for about 20 s. Soil macroaggregates (on top of the sieve) and soil microaggregates (within the collection container) were collected as sum and the weights were recorded. We applied dry-sieving in order to also obtain not-water stable macroaggregates as well, as wetting of the soil and subsequent wet-sieving would have led to a break-down of not-water stable soil macroaggregates. In addition, concomitant drying (either air- or freeze-drying) would have increased the risk of more severe sample alterations like a redistribution of elements between size-fractions compared to dry-sieving [37]. Therefore, dry-sieving in the here described manner was the most gentle way to separate macro- and microaggregates without significant sample alterations.

It is assumed that most of the particles isolated are aggregates; however, it cannot be excluded that also single primary particles are also present in the microaggregate size fraction. One possible method to separate these particles is density fractionation [38]. However, this additional fractionation procedure can result in an artificial alteration of the soil structure and nutrient composition on the surface of the aggregates. Furthermore, surfaces of primary particles also can be coated by short-range ordered oxyhydroxides like iron (hydr)oxides or organic matter [9] and thus can also adsorb anions like phosphates on their surface when pH is below the point of zero charge of the surface coatings [39]. Hence, particles can be also seen as simple aggregates. Therefore, we have consciously integrated these particles into our samples and, thus, when referring to microaggregate samples here we also include primary particles <250 μm.

Always three technical replicates of the independent field replicates were analyzed for their chemical composition. Soil microaggregate samples (<250 μm) were analyzed for their pH using a 1:2.5 soil:solution (w/v) suspension in 0.01 M CaCl_2_ solution, total elemental composition (P_,_ S, Ca, Mg, Fe) concentrations were determined after microwave assisted digestion of 150 mg sample with 0.7 mL HNO_3_ and 2 mL HCl using an inductively coupled plasma-optical emission spectroscopy (ICP-OES; Thermo Fisher iCAP 7600). The soil was free of inorganic C; thus, total C and N analyses were done using dry combustion followed by heat conductivity detection of the released trace gases (vario MICRO cube, Elementar, Hanau, Germany).

A sequential fractionation was performed according to Tiessen and Moir [40] with slight modifications. The modifications were that the inorganic P (Pi) concentration in the extracts was determined colorimetrically with the molybdate blue method as described by [41] using a UV-spectrometer (Vario EL, Elementar Analysensysteme, Hanau, Germany). Briefly, samples were subsequently treated with solutions of rising extractant strength: Resin (Resin-P), 0.5 M NaHCO_3_ (NaHCO_3_-P), 0.1 M NaOH (NaOH-P), concentrated HCl (HCl-P), and Aqua regia (Residual-P). The resin P (Resin-P) is representing the easily exchangeable and soil solution P, inorganic (NaHCO_3_-P_i_) and organic (NaHCO_3_-P_o_) bicarbonate P is representing the labile (adsorbed) inorganic and organic P as well as microbial P, inorganic (NaOH-P_i_) and organic P (NaOH-P_o_) hydroxide is representing moderately labile inorganic and organic P sorbed and/or fixed by aluminum- and iron (hydr)oxide (accessory minerals) and P in humic and fulvic acids potentially bioavailable. The concentrated HCl-P fractions represent insoluble (stable) P associated with Ca and Mg minerals being in occluded or non-occluded forms. Total P in the extracts was measured using ICP-OES and the concentration of Po was estimated by subtracting Pi from total P.

In the sequential extracts as described for P analyses above (Resin, 0.5 M NaHCO_3_, 0.1 M NaOH, concentrated HCl, and aqua regia digestion) the total S concentration was also determined using ICP-OES analyses. In the resin extract mainly soluble S and simple organic esters are extracted that are short-term plant available [28]. The 0.5 M NaHCO_3_ solution is known to generally extract weakly adsorbed S [27] and also may dissolve organic S forms [42,43]. The 0.1 M NaOH solution extracts the organic matter bound S and S being more strongly associated to Fe- and Al-hydrous oxides as the adsorption of anions to most minerals surfaces is minimal at alkaline pH values ([44] and references therein). That would meet medium- to long-term plant nutrient availability [27,28], whereas within the HCl extract the S associated with Ca compounds, Fe hydrous oxides, as well as acid hydrolysable organic S and some inorganic S occluded in crystalline Fe oxides are determined representing the residual fraction [27,45].

### 2.3 Py-FIMS

Temperature-resolved Py-FI mass spectroscopy was performed by the research group of P. Leinweber at the University of Rostock according to [25] on three replicates of the microaggregate size fraction of the topsoil control sample and of the topsoil 10 and 24 month after clear-cut. The replicates were averaged after the measurement. In short, in a double-focusing mass spectrometer (Finnigan MAT 731, Germany) equipped with a direct inlet 0.5 mg sample was heated in 10°C steps in a vacuum of 10^−4^ Pa from 110 to 700°C over a time period of 15 minutes. In order to remove residues of pyrolysis products the emitter was flash heated between the scans. During the analysis, 60 spectra were recorded in the mass range *m/z* 15 to 900. We obtained Py-FIMS spectra by averaging the replicate measures and summing up the mass signals over the whole temperature range and plotting them against the relative abundance in % total ion intensity (TII). Thermograms were obtained by plotting the TII being normalized to the sample weight against the pyrolysis temperature. For spectra interpretation we used marker signals (*m/z*) that are assigned and grouped to eleven relevant compound classes as described in literature [25]. Volatilized matter (VM) was calculated by mass loss of the sample due to pyrolysis in wt.%.

Characteristic thermograms for individual compound classes were obtained by plotting the average ion intensities against the pyrolysis temperature. These plots visualize the thermal energy required for volatilization of individual biomarkers of SOM also hinting to their stability.

The hexose to pentose ratio (H:P) was calculated by the sum of ion intensities of masses of hexoses (galactose, mannose, *m/z* 126, 127, 144, 145, 162, 163) and divided by the sum of ion intensities of masses of pentoses (arabinose, xylose, *m/z* 114, 115, 132, 133) [46]. Generally, hexoses represent microbial derived materials while pentoses represents plant-derived materials [47], the hexose to pentose ratio can thus be used as proxy to determine the share of microbial SOM [48,49]. The thermal stability was calculated as ratio of the measured ion intensities ≥410°C proportional to the total ion intensity (in 10^6^ counts mg^-1^) of the sample and is expressed as percentage (%).

### 2.4 XANES spectroscopy

For bulk XANES analyses samples from individual field replicates were pooled to yield homogenized composite samples. Bulk P and S *K*-edge XANES spectra were recorded at the Soft X-ray Micro-characterization Beamline (SXRMB) at the Canadian Light Source synchrotron, Saskatoon, Canada [50]. The air-dried and homogenized samples were spread as thin film onto a double-sided carbon tape and mounted onto a copper sample holder. The spectra were recorded in fluorescence yield mode (samples) and total electron yield mode (reference standards). Spectra were recorded in range 2130 to 2200 eV (P *K*-edge XANES) and 2435 to 2540 eV (S *K*-edge XANES) using a dwell time of 4 s (samples) and 1 s (reference compounds), respectively. The absolute energy was calibrated using the Ar *K*-edge around 3205 eV; this means that the main peak position of Al phosphate (AlPO_4_) was around 2152.9 eV. At least two scans were recorded for each sample. Subsequent data treatment such as normalization to the intensity of the incident beam, spectra averaging, background correction, normalization as well as linear combination fitting (LCF) were done using ATHENA (Demeter 0.9.24) [51]. For P XANES spectra a LCF was done on averaged, normalized spectra in the energy range between -12 eV and +30 eV relative to the E_0_ for all possible binary to quaternary combinations of the following reference standards: Ca_10_(PO_4_)_6_ (OH)_2_, Ca(H_2_PO_4_)_2_·H_2_O, CaHPO_4_, CaHPO_4_2H_2_O, amorphous calcium-phosphate, Mg_2_O7P_2_, MgHPO_4_·3H_2_O, AlPO_4_, P adsorbed on gibbsite, P adsorbed on goethite, P adsorbed on ferrihydrite, and C_6_H_18_O_24_P_6_·Na·yH_2_O (phytic acid = proxy for P_o_).

For S XANES spectra the LCF was done on averaged, normalized total electron yield spectra in the energy range between -20 eV and +35 eV relative to the E_0_ for all possible binary to senary combinations of the following reference standards: Disulfide = Dibenzyl disulfide, pyrite, Thiol = L-cysteine, Thiophene = Dibenzothiophene, Thioether = L-methionine, Sulfoxide = phenylsulfoxide, Sulfonate = methanesulfonate, Sulfone = saccharin, sulfate = iron(III) sulfate, and iron(II) sulfate heptahydrate. The r-factor values were used as goodness-of-fit criteria and their significant difference between fits were evaluated using the Hamilton test [52] (p <0.05) with the number of independent data points calculated by Athena estimated as data range divided by core-hole lifetime broadening. If by adding a further reference compound to a fit the R-factor was not significantly better according to the Hamilton test, the fit with the lower number of reference compound was chosen. If fits with the same number of reference compounds were not significantly different from each other, fit proportions were averaged and standard deviations are shown.

### 2.5 Statistical analyses

Statistical analyses were performed using IBM SPSS 22 (IBM SPSS Statistics V. 22.0, 2013, IBM Inc.). We tested the P and S data for normal distribution by Shapiro-Wilk test (P < 0.05) and for homogeneity of variances by Brown-Forsythe test (P < 0.05). We considered samples from different depth as paired samples. We performed a One-Way ANOVA with the factor sampling time. If significant differences occurred, we used the Tukey HSD test for post-hoc separation of means (P < 0.05). To test for the effect of depth within one sampling time we performed paired t-tests for each year and depths separately (P < 0.05). Linear regression analysis was performed to elucidate the relationship between the thermal stability and the ratio of microbial versus plant-derived carbohydrates of microaggregate topsoil samples.

## 3 Results

### 3.1 General soil parameters

Based on the analysis results presented in Siebers et al., [10], e.g., the slightly differing elemental stocks and especially the missing temporal trend of P on the native site, we considered all non-clear-cut sites as controls. Therefore, we pooled all data before the clear-felling event, namely the native site 2013, 2014, and 2015 and the clear-cut site 2013 right before the clear-cut, to represent the control value.

The proportions of mass yield of the microaggregate size fraction before clear-cut (control) were not significantly different in the topsoil than in the subsoil, whereas the pH value was lower in the topsoil (bulk soil and microaggregate size fraction) than in the subsoil (Tables 1 and 2). Topsoil total C and N contents as well as C:N ratios were generally higher than in subsoils for both bulk soil and the microaggregate size fraction; however, soil microaggregates tend to be slightly enriched in C and N. There were no clear differences in total elemental contents between bulk soil and the microaggregate size fraction except for P, being generally (except for the subsoil 10 month after the clear-cut) enriched in the microaggregate size fraction for both, top- and subsoils.

**Table 1:** Bulk soil texture, pH_CaCl2_ values and elemental composition given as mean ± standard deviation (SD) (n = 3). Total C and N concentrations were determined by dry total combustion, all other total elemental concentrations were determined by microwave-assisted digestion in *aqua regia*. Different lowercase letters indicate significant (*P<0*.*05*) differences with time for bulk samples, respectively, whereas different uppercase letters indicate significant differences of bulk samples at the respective sampling time.

Sampling year	Treatment	Texture [%]			pH _CaCl2_	C	N	C:N	P	S	Ca	Mg	Fe
		Clay	Silt	Sand	[–]	[g kg^-1^]	[g kg^-1^]	[–]	[mg kg^-1^]			[g kg^-1^]	
**Bulk topsoil**													
2013	**Native**	27.8±0.8	57.2±1.4	15.0±0.6	3.4±0.1 ^A^	63.4±4.2 ^A^	3.3±0.5 ^A^	19.2±0.9 ^A^	580±22	405±18 ^A^	240±31 ^a^	2.4±0.2	34.4±0.7 ^A^
2014					3.5±0.1 ^A^	49.6±3.5 ^A^	2.4±0.1 ^A^	20.7±2.4 ^A^	589±31	430±19	200±24 ^aA^	2.5±0.4	33.5±0.9 ^A^
2015					3.6±0.2	58.7±2.4 ^A^	3.4±1.0 ^A^	17.3±0.7 ^A^	620±29 ^A^	370±35	311±12 ^b^	2.2±0.1	33.6±1.2 ^A^
**Bulk subsoil**		25.4±3.3	54.7±1.3	19.9±4.3									
2013	**Native**				4.1±0.1 ^aB^	9.3±0.1 ^B^	1.2±0.1 ^B^	7.8±0.1 ^B^	561±54	324±53 ^B^	282±15	2.4±0.1	39.4±2.4 ^aB^
2014					4.2±0.2 ^aB^	9.9±0.2 ^B^	1.2±0.2 ^B^	8.3±0.6 ^B^	495±43	402±43	324±41 ^B^	2.1±0.2	42.1±1.3 ^aB^
2015					3.8±0.1 ^b^	9.2±0.4 ^B^	1.3±0.1 ^B^	7.1±0.5 ^B^	502±34 ^B^	367±21	334±27	1.9±0.1	48.3±1.9 ^bB^
**Bulk topsoil**													
2013	**Clear-cut**	24.4±1.9	59.7±0.8	15.9±2.6	2.9±0.1 ^aA^	57.4±3.5 ^aA^	3.3±1.2 ^aA^	17.4±0.8 ^A^	543±18 ^a^	420±12	370±31 ^A^	4.3±0.4 ^aA^	32.1±0.6 ^aA^
2014					3.5±0.3 ^bA^	76.7±0.0 ^bA^	4.2±0.1 ^bA^	18.5±0.1 ^A^	590±10 ^b^	410±20	400±30	5.1±0.3 ^aA^	40.3±0.8 ^b^
2015					3.6±0.1 ^bA^	97.8±5.8 ^cA^	6.1±0.5 ^cA^	16.2±2.2 ^A^	634±12 ^c^	409±32	378±18 ^A^	2.1±0.4 ^b^	38.3±0.9 ^bA^
**Bulk subsoil**													
2013	**Clear-cut**	27.7±0.3	53.5±4.9	18.9±4.6	4.2±0.1 ^B^	9.1±0.2 ^B^	1.3±0.0 ^B^	7.0±0.2 ^aB^	523±27 ^a^	386±19 ^a^	305±18 ^aB^	2.2±0.1^B^	45.2±1.1 ^aB^
2014					4.1±0.1 ^B^	8.6±0.2 ^B^	1.1±0.0 ^B^	7.8±0.2 ^aB^	584±59 ^a^	438±46 ^a^	365±29 ^b^	2.7±0.3 ^B^	38.2±2.4 ^b^
2015					4.1±0.0 ^B^	18.9±0.3 ^B^	1.9±0.1 ^B^	9.9±0.2 ^bB^	630±30 ^b^	510±70 ^b^	308±18 ^aB^	2.1±0.2	33.6±1.9 ^cB^

Samples were taken 10 and 24 month after clear-cut; n.d.: not determined as sample was already soil microaggregate fraction

**Table 2:** Soil microaggregate pH_CaCl2_ values and elemental composition of pooled samples without clear-cut (control) given as mean ± standard deviation (SD) (n = 3). Total C and N concentrations were determined by dry total combustion, all other total elemental concentrations were determined by microwave-assisted digestion in *aqua regia*. Different lowercase letters indicate significant (*P<0*.*05*) differences with time for microaggregate samples, respectively, whereas different uppercase letters indicate significant differences of microaggregate samples at the respective sampling time.

	Mass yield microaggregates	pH_CaCl2_	C	N	C:N	P	S	Ca	Mg	Fe
	[%]	[–]	[g kg^-1^]	[g kg^-1^]	[–]	[mg kg^-1^]			[g kg^-1^]	
**Microaggregates topsoil**										
Control	30.9±1.7 ^a^	3.7±0.2 ^aA^	65.1±1.4 ^aA^	3.6±1.1 ^aA^	17.9±3.1 ^a^	635±41 ^a^	424±38 ^a^	234±8 ^a^	3.2±0.1 ^aA^	41.4±0.8
10 month	36.1±0.8 ^bA^	3.4±0.1 ^bA^	82.3±8.0 ^bA^	4.5±0.2 ^bA^	18.4±1.2 ^a^	644±8 ^abA^	404±8 ^a^	390±30 ^bA^	4.4±0.2 ^bA^	37.8±0.9
24 month	36.7±0.7 ^b^	3.5±0.0 ^cA^	86.8±0.4 ^bA^	4.0±0.1 ^cA^	21.6±2.1 ^b^	729±12 ^b^	507±23 ^b^	230±20 ^a^	2.3±0.1 ^cA^	41.9±0.7
**Microaggregates subsoil**										
Control	28.7±2.3 ^ab^	4.2±0.1 ^B^	11.8±1.5 ^aB^	1.5±0.4 ^aB^	8.0±0.9 ^a^	517±117	442±130	190±20	7.4±0.1 ^aB^	39.8±0.7
10 month	27.1±2.8 ^aB^	4.1±0.0 ^B^	8.3±1.1 ^bB^	1.2±0.2 ^aB^	6.7±1.4 ^b^	525±68 ^B^	367±16	150±30 ^B^	8.8±0.2 ^aB^	43.3±1.2
24 month	33.7±2.2 ^b^	4.1±0.0 ^B^	36.5±1.2 ^cB^	3.4±0.6 ^bB^	15.4±4.2 ^c^	699±73	435±45	210±30	3.9±0.1 ^bB^	41.2±1.4

Samples were taken 10 and 24 month after clear-cut.

Mass proportions of soil microaggregate size fraction significantly increased after the clear-cut, being more pronounced in the topsoil than in the subsoil (Table 3). Clear-cutting also significantly increased bulk topsoil pH, while pH was decreased in the topsoil microaggregate size fraction. Generally, C and N significantly increased 24 month after the clear-cut for all bulk and microaggregate size fraction samples being more pronounced for the top- than for the subsoil. In the bulk samples the C:N ratio kept constant with time, whereas in the top- and subsoil samples of the soil microaggregate size fraction the C:N ratio significantly was increased after 24 month post clear-cut. A significant increase in bulk (P, Fe) and microaggregate size fraction (P) of the topsoil and S in the microaggregate size fraction of the topsoil was also observed. By contrast, concentrations of Ca and Mg did not change 24 month after the clear-cut except for the Mg concentrations in the microaggregate size fraction which decreased in both the top and subsoil (Table 3).

**Table 3:** Proportions of ion intensity of different compound classes (%TII) and total ion intensity in parentheses (TII counts mg^-1^) of the microaggregate size fraction of topsoil samples of the control without clear-cut, 10, and 24 month after clear-cut. Values followed by the same letters within a compound class were not significantly different (P < 0.05, n = 3).

	Control	10 month	24 month
	TII from compound classes		
CHYDR	1.7^a^ (9.7)	2.8^b^ (18.1)	1.6^a^ (14.6)
PHLM	1.7^a^ (9.7)	4.4^b^ (29.1)	3.1^a^ (28.8)
LDIM	3.0^a^ (17.2)	4.3^ab^ (28.3)	4.6^b^ (42.9)
LIPID	3.9^a^ (22.5)	6.7^b^ (44.1)	7.1^c^ (66.1)
ALKYL	7.8^a^ (45.3)	8.6^a^ (56.5)	8.3^a^ (77.5)
NCOMP	0.7^a^ (3.9)	0.9^b^ (6.1)	0.6^c^ (5.3)
STEROL	9.8^a^ (57.0)	8.8^b^ (57.5)	14.3^c^ (133.5)
PEPTI	1.9^a^ (11.3)	2.6^b^ (16.9)	1.8^a^ (16.6)
SUBER	0.6^a^ (3.2)	0.4^b^ (2.7)	0.4^b^ (3.8)
FATTY	2.3^a^ (13.6)	1.5^b^ (10.1)	1.2^c^ (11.0)
*m/z* 15…56	1.0^a^ (5.7)	1.3^b^ (8.6)	0.8^c^ (7.6)

CHYDR, carbohydrates; PHLM, phenols and lignin monomers; LDIM, lignin dimers; LIPID, long-chained hydrocarbons; ALKYL, alkylaromatics; NCOMP, N-containing non-peptidic compounds; STEROL, sterols; PEPTI, peptides; SUBER, suberin; FATTY, free fatty acids C16-C34. Values followed by the same letters within a line were not significantly different (P < 0.05).

### 3.2 Pyrolysis-field ionization mass spectrometry (Py-FIMS)

**Fig 1:**
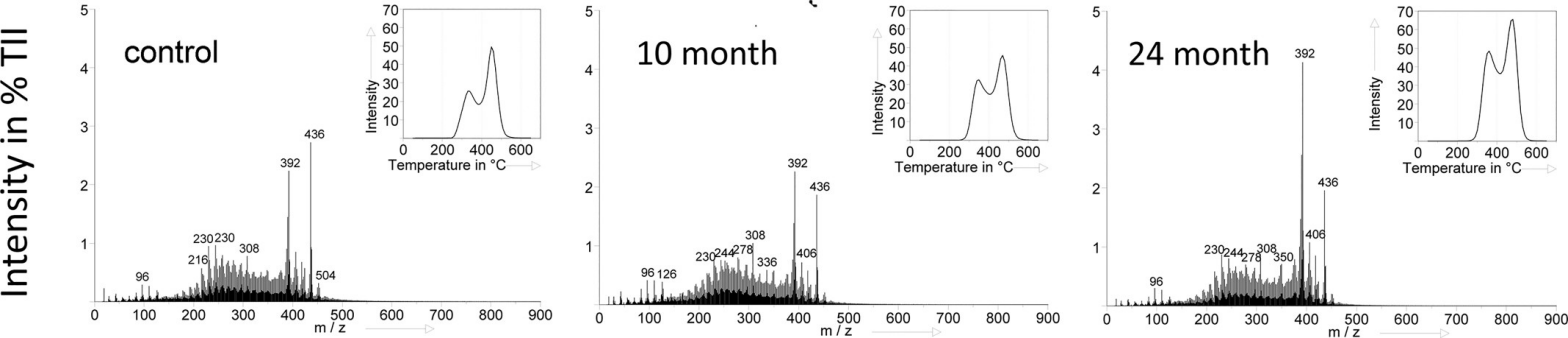
Thermograms of total ion intensity (%TII) (upper right) and summed averaged pyrolysis-field mass spectra of samples for the microaggregate size fraction of the control without clear-cut, 10, and 24 month after clear-cut.

All Py-FIMS spectra were dominated by *m/z* 392 (C_29_H_44_ and C_28_H_56_, unsaturated ethylcholestanes) and *m/z* 436 (C_30_H_44_O_2_, monomeric syringyl units), while the relative intensity of *m/z* 392 was lower than *m/z* 436 in the control sample and increased with increasing time after the clear-cut (Fig 1). Furthermore, all spectra showed characteristic marker *m/z* signals for carbohydrates (*m/z* 96), lipids and alkanes (*m/z* 230), and all samples after clear-cut were characterized by a higher intensity of *m/z* 308 (C_22_H_44_, alkenes) than the control sample. Some spectra also showed *m/z* 126 (carbohydrates), 216 (hexose), 244 (lipids, alkanes), 278 (neophyt-adiene), 336 (styrene-d8 homo-trimers), 350, and 406 (long-chained lipids) (Fig 1).

Assignment and grouping of marker signals to important eleven compound classes of SOM revealed changes in the relative intensity (% detected total ion intensity) and in the absolute intensity (counts mg^-1^). Highest proportion and absolute intensities among all compound classes were found for sterols and alkylaromatics (Table 3). Ten month after clear-cut proportions and total intensities of CHYDR, PHLM. LDIM, LIPID, ALKYL, NCOMP, and PEPTI increased, whereas STYROL and FATTY decreased. With increasing time after clear-cut the proportions of lignin dimers, lipids, and sterols significantly increased, whereas CHYDR, PHLM, NCOMP, PEPTI, and FATTY significantly decreased (Table 3).

The Py-FIMS spectra also indicated a hexose to pentose ratios of 3.47±0.21^a^ (control), 3.13±0.05^b^ (10 month), and 3.38±0.07^a^ (24 month) (different letters indicate significant differences among sampling times at P < 0.05). The ratio significantly decreased (P < 0.05) 10 month after clear-cut and then increased again approximating the control value.

In Fig 1 the total ion intensity (TII) thermogram (see upper right insert) for all samples were bimodal in shape indicating a thermolabile (volatile <400°C) and thermostable (volatile ≥410°C) proportion of SOM. The volatilization maxima were around 330°C and 450°C. Ten month after clear-cut the ratio between thermolabile and thermostable proportions became more narrow. After 24 month the ratio did not change compared to 10 month; however, the total intensity increased.

**Fig 2:**
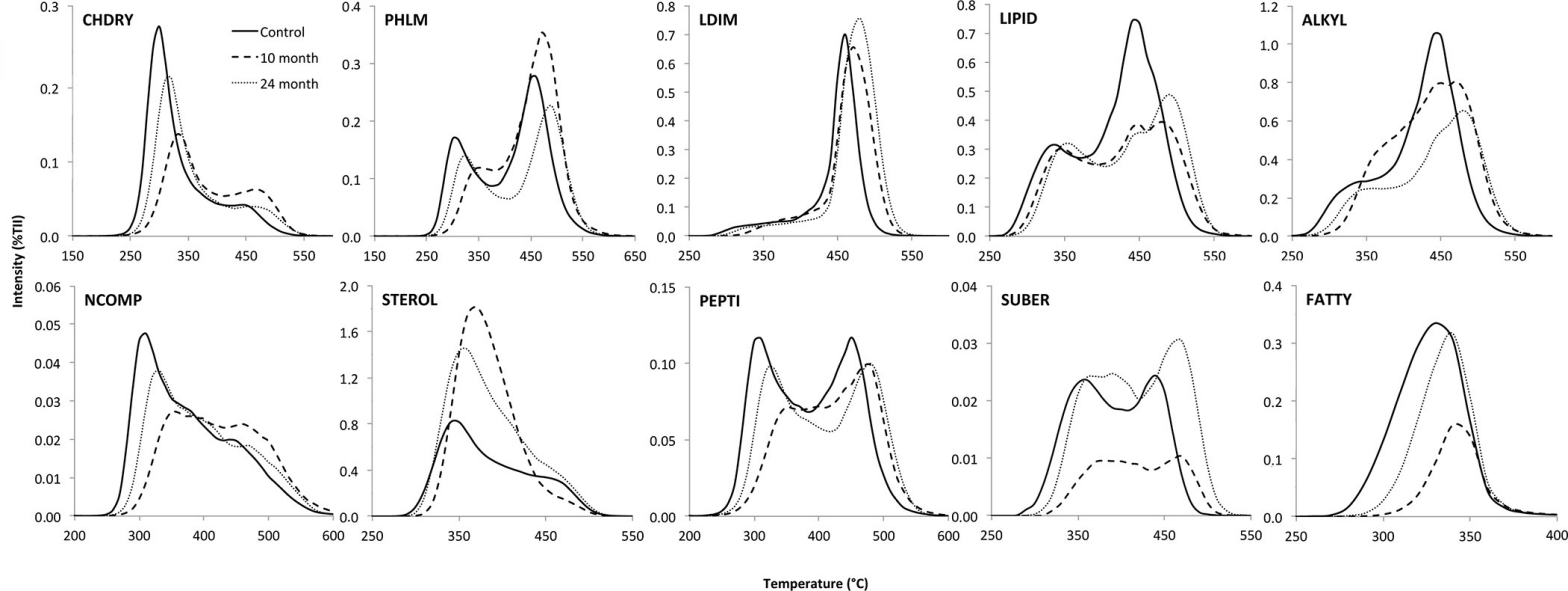
Pyrolysis field thermogram of the compound classes CHYDR, carbohydrates; PHLM, phenols and lignin monomers; LDIM, lignin dimers; LIPID, long-chained hydrocarbons; ALKYL, alkylaromatics; NCOMP, N-containing non-peptidic compounds; STEROL, sterols; PEPTI, peptides; SUBER, suberin; and FATTY, free fatty acids C16-C34 normalized to the total ion intensity (TII) of samples for the control without clear-cut, 10, and 24 month after clear-cut. Peaks at higher temperatures indicate higher thermal stability.

The thermal volatilization curves of individual compound classes were generally bimodal in shape (Fig 2), except for the compound classes LDIM (lignin dimers) and FATTY (free fatty acids). While the thermal volatilization curve of LDIM hardly changed in the course of the clear-cut, the thermal volatization curves of the other compound classes were strongly affected. Generally, the greatest change in curve progression was visible 10 month after the clear-cut, which approached the control again 24 months later. Exemptions to this were PHLM (phenols and lignin monomers), LIPID (lipids, alkanes, alkenes, and alky monoesters), and ALKYL (alkylaromatics). The compound classes like CHDRY (carbohydrates) and NCOMP (N-containing compounds) showed a reduction of the thermolabile proportions with clear-cut, whereas the thermal bimodal volatilization curves of the compound class LIPID showed a shift of the both volatilization maxima towards higher temperatures. This shift was more pronounced for the high temperature volatilization peak. There was a minor shift of the thermal volatilization curve towards higher temperatures for ALKYL class with time after clear-cut, while the proportions of the high temperature peak decreased. The volatilization curve of STEROL (sterols) shifted from a bimodal shape towards monomodal shape of higher relative intensity peaking around 360 to 375°C 10 month after clear-cut. Twenty-four month after clear-cut the more asymmetrical volatilization curve of STEROL showed a maximum being slightly shifted towards lower temperature and was reduced in relative intensity (Fig 2). A reduction of the thermolabile proportions were also observed for the compound class PEPTI (peptides) and SUBER (suberin), with and initial reduction of both thermolabile and thermostable peaks, being more pronounced for the thermolabile peak, and a generally shift of the volatilization curve towards higher temperatures. However, 24 month after clear-cut the curve approached the progression of the control-curve again.

**Fig 3:**
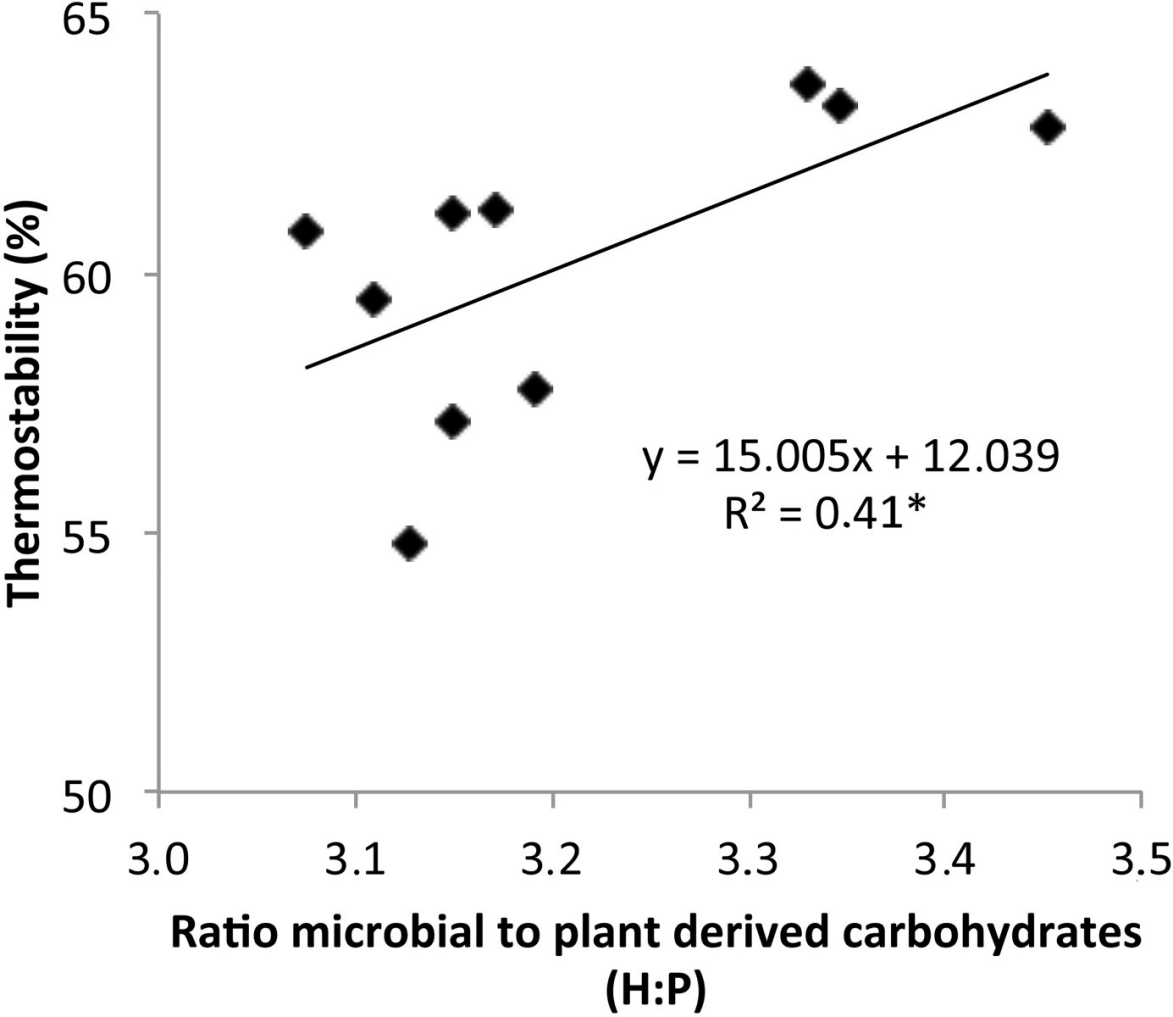
The correlation of thermal stability (calculated as ratio of the measured ion intensities ≥410°C proportional to the total ion intensity (in 10^6^ counts mg^-1^) of the sample and expressed as percentage (%)) with the ratio of microbial versus plant-derived carbohydrates (H:P, hexose:pentose ratio) of microaggregate size fraction of the topsoil samples. The level of significance is given by superscript asterisks: *P < 0.05.

The general thermal stability of SOM in microaggregates significantly increased with increasing time after clear-cut being 57.3±1.9^a^ (control), 61.0±0.2^b^ (10 month), and 63.2±0.4^c^ (24 month) (different letters indicate significant differences among sampling times at P < 0.05). The thermal stability of all samples significantly positively correlated to the hexose to pentose ratio (P = 0.047; n = 10, four replicate measurements for the control site, and three each for the 10 and 24 month after the clear-cut samples) (Fig 3).

### 3.3 P and S sequential fractionation

Total P concentrations within the microaggregate size fraction were generally higher in topsoils than in subsoils, although this effect was significant only 10 month after the clear-cut. When considering the single P fractions, P concentrations were also mainly higher in topsoils, although this effect was significant only in few cases. There were significantly higher P concentrations in the HCl fraction (top- and subsoil), as well as P_t_, (topsoil) and significantly lower Po_OH_ (subsoil) concentrations.

For the control, the relative distribution of P among the sequential P fractions was more or less similar for top- and subsoil. After the clear-cut this was different with lower proportions of labile P_Resin_ with increasing time in top- as well as subsoils. Change in the proportions and concentrations of moderate labile (i.e., Pi_OH_ and Po_OH_) were more pronounced in the subsoil than in the topsoil. Ten month after clear-cut proportions of Pi_OH_ first decreased relative to the control and then increased again relative to the control after 24 month. The opposite was true for the Po_OH_ (Table 4).

**Table 4:** Concentrations (mg kg^-1^) ± standard deviation of sequentially extracted phosphorus (P) fractions and proportions of total P (P_t_) in parenthesis of the control without clear-cut, 10, and 24 month after clear-cut. Different lowercase letters indicate significant (*P<0*.*05*, n = 3) differences with time for top- or subsoil samples, respectively, whereas different uppercase letters indicate significant differences of top- and subsoil samples at the respective sampling time.

	P_Resin_	Pi_HCO3_	Po_HCO3_	Pi_OH_	Po_OH_	P_HCl_	P_t_
	[mg kg^-1^]						
**Topsoil**							
Control	73±33 (11)	14±5 (2) ^A^	21±6 (3) ^A^	124±38 (19)	177±50 (27)	162±36 (25) ^a^	635±41 ^a^
10 month	67±6 (10)	14±1 (2) ^A^	24±0 (3) ^A^	85±2 (13)	142±69 (22)	136±36 (21) ^a^	644±8 ^abA^
24 month	55±5 (7)	15±1 (2)	27±3 (3)	154±10 (21) ^A^	168±46 (23) ^A^	235±2 (32) ^b^	729±12 ^b^
**Subsoil**							
Control	64±26 (12)	7±5 (1) ^B^	10±8 (1) ^B^	128±52 (24)	152±69 (29) ^a^	162±36 (31) ^a^	517±117
10 month	51±0 (9)	3±0 (0) ^B^	2±1 (0) ^B^	92±2 (17)	226±33 (43) ^a^	148±7 (28) ^a^	525±68 ^B^
24 month	40±1 (5)	9±0 (1)	16±3 (2)	212±30 (30) ^B^	45±15 (6) ^bB^	242±3 (34) ^b^	699±73

The chemical fractionation appeared to recover between 66 to 101% of the total S in the microaggregate size fraction (Table 5). Total S concentrations were comparable in top- and subsoils. We were not able to distinguish between organically and inorganically bound S as was done for P. The highest S concentrations were found in the S_Resin_ fraction, whereas the other fractions had comparable S concentrations. Concentrations of S in NaOH- and HCl-fractions were significantly higher in the topsoil than in the subsoil for the control.

**Table 5:** Concentrations (mg kg^-1^) ± standard deviation of sequentially extracted sulfur (S) fractions and proportions of total S (S_t_) in parenthesis of the control without clear-cut, 10, and 24 month after clear-cut. Different lowercase letters indicate significant (*P<0*.*05*; n = 3) differences with time for top- or subsoil samples, respectively, whereas different uppercase letters indicate significant differences of top- and subsoil samples at the respective sampling time.

	S_Resin_	S_HCO3_	S_OH_	S_HCl_	S_t_
	[mg kg^-1^]				
**Topsoil**					
Control	118±39 (28) ^a^	60±20 (14)	65±10 (15) ^A^	58±10 (14) ^A^	424±38 ^a^
10 month	134±82 (33) ^ab^	57±9 (14) ^A^	58±13 (14)	56±18 (14)	404±8 ^a^
24 month	239±8 (47) ^b^	65±10 (13)	56±13 (11)	42±16 (8)	507±23 ^b^
**Subsoil**					
Control	138±60 (31) ^ab^	60±16 (14) ^a^	55±11 (12) ^aB^	40±11 (9) ^abB^	442±130
10 month	71±2 (19) ^a^	86±10 (24) ^abB^	81±10 (22) ^b^	62±3 (17) ^a^	367±16
24 month	234±21 (54) ^b^	102±3 (24) ^b^	69±5 (16) ^ab^	29±4 (7) ^b^	435±45

Topsoil S_Resin_ and total S concentrations significantly increased only in samples 24 month after clear-cut. In the subsoil there were also significant increases in S concentrations in the NaHCO_3_-, and the NaOH-fraction, whereas the S_HCl_ concentrations decreased 24 month after the clear-cut. Similar to the significant changes of absolute S concentrations after the clear-cut, the S proportions in the fractions in relation to total S also in- or decreased. Thus, the relative distribution of S among sequential S fractions was affected by the clear-cut (Table 5).

### 3.4 P and S speciation

Linear combination fitting of the P *K*-edge spectra of the soils collected before the clear-cut indicated that topsoil microaggregates were dominated by P sorbed to ferrihydrite (64%) along with lower proportions of P sorbed to gibbsite (18%) and organic P (19%) before the clear-cut (Table 5). Proportions of P sorbed to ferrihydrite were same between topsoil and subsoil, but contributions of Al-associated P were higher (5% points) and contribution of organic P lower (6% points) in the subsoil (Table 6). In the course of clear-cutting the P speciation in the topsoil revealed no contribution of Al-associated P (P sorbed to gibbsite) to P speciation anymore, whereas the proportion of organic P almost doubled to 32% 10 month after the clear-cut. Twenty-four month after clear-cutting topsoil P speciation in the microaggregate size fraction shifted again to the lower organic P contribution of the control, while proportions of Fe-associated P increased with less clear differentiation between P sorbed to ferrihydrite or goethite. In the subsoil, clear-cutting only slightly affected P proportions, with decreasing contributions of P sorbed to gibbsite with increasing time after the clear-cut and slightly increased organic P proportions 24 month after clear-cut being comparable to proportions of the topsoil microaggregate size fraction.

**Table 6:** Results of linear combination fitting of P *K*-edge XANES spectra of forest soil microaggregates size fraction of the control without clear-cut, 10, and 24 month after clear-cut. Values in brackets shown the standard deviation of averaged fit proportions in the case when fits were not significantly different to each other (Hamilton P ≤ 0.05) based on the r-factor.

	P sorbed to Gibbsite	P sorbed to Ferrihydrite	P sorbed to Goethite	P_o_	r-factor
	%				
**Topsoil**					
Control	18	64		19	0.003280
10 month		68		32	0.003894
24 month	6±6	57±9	18±18	19±3	0.003711
**Subsoil**					
Control	23	63		13	
10 month	21	69		10	0.004939
24 month	18	65		17	0.003051

We grouped the different S reference compounds and associated S oxidation states into three major groups according to [53] namely (i) reduced S forms (disulfides, thiol, thiophenes), (ii) intermediate S forms (sulfoxide, solfone, sulfonate), and highly oxidized S forms (sulfate). The fitting of the S *K*-edge spectra indicated that highly oxidized-S (46%) was the major S-form in topsoil microaggregates of the control, followed by reduced S forms (32%), and the lowest contribution made up the intermediate S forms (21%) (Table 7). Generally, the highly oxidized and intermediate S forms were more dominant than the reduced-S forms.

**Table 7:** Results of linear combination fitting of S *K*-edge XANES spectra of forest soil microaggregate size fraction of the control without clear-cut, 10, and 24 month after clear-cut. Reference substances: disulfide = Dibenzyl disulfide, thiol = L-cysteine, sulfide = hexakis(benzylthio)benzene, thiophene = dibenzothiophene, sulfoxide = phenylsulfoxide, Thioether = L-methionine, sulfone = dibenzothiophene sulfone, sulfonate = methanesulfonate, sulfate = calcium sulfate dehydrate and oxidation state of S in these compounds. Values in brackets shown the standard deviation of averaged fit proportions in the case when fits were not significantly different to each other (Hamilton P ≤ 0.05) based on the r-factor.

	Reduced S				Intermediate S			Highly oxidized S	
	Disulfide	Thiol	Thiophene	Thioether	Sulfoxide	Sulfone	Sulfonate	Sulfate	r-factor
	(-1 to -2)	(-2)	(-2)	(-2)	(0)	(+2)	(+4)	(+6)	
	%								
**Topsoil**									
Control	10		18	4	6	8	7	46	0.00873
10 month	11		26			15	14	34	0.01066
24 month	5±5	9±9	17 ±4		12±0	10±0		47±0	0.01730
**Subsoil**									
Control							1	99	0.035811
10 month		2±4	2±4	2±3		2±4	2±4	91±0	0.029000
24 month								100	0.033603

In the topsoil microaggregate size fraction clear-cutting further shifted the proportions towards intermediate-S forms with increasing proportions of sulfone and sulfonate, while thiophenes also increased by 12% points on the expense of sulfoxide and sulfate. After 24 month proportions of sulfone decreased again to and sulfonate in the topsoil microaggregate size fraction were not present anymore while sulfate increased again and proportions of reduced S such as disulfides thiophenes decreased. For the subsoil microaggregate size fraction the fitting of the S *K*-edge spectra revealed that sulfate accounted for the main proportions of S-forms constituting between 91 to 100% for all sampling times. However, 10 month after clear-cut there were minor contributions of reduced or intermediate S forms (Table 7).

## 4 Discussion

### 4.1 Influence of the clear-cut on organic matter composition

The observed higher proportions of the macroaggregate size fraction (63 to 73%) over the microaggregate size fraction hint to a well-developed soil structure of the forest soil. The increase in the proportion of the microaggregate size fractions (Table 2) after the clear-cut can be explained by the disruption of the soil structure due the destabilization of soil macroaggregates >250 μm, which was described also in other studies [3,4,15,16]. Soil aggregates are to a great extent stabilized by SOC [9,54], and it is a generally accepted fact that clear-cuts foster decomposition and losses of SOC [55], especially of the strongly mineral-bound and stable C pool [56]. Consequently, physical weakening of macroaggregates and microbial decomposition of aggregate-associated SOC might have led to macroaggregate disintegration [57] being indicated by increasing microaggregate size fraction mass yields. Siebers et al. [10] found that a reduced stability and breakdown of aggregates foster decomposition of exposed SOM and increased P accessibility and microbial P turnover in the soil microaggregate size fractions after the clear-cut.

However, in our samples decomposition of SOM, as indicated by a reduced organic C concentration, was not directly observed, instead C concentrations increased in bulk soils and microaggregates size fractions after clear-cut. This can be explained by increased addition, incorporation, and decomposition of clear-cut slash residues [58] or root remains in the soil. It is reasonable that such extra input in SOC can mask a possible clear-cut induced increased decomposition of SOC, as suggested by Siebers et al. [10]. Nevertheless, the increase in SOC was likely not directly linked to clear-cut induced changes in aggregate stability.

Along with an increase in organic C the total N concentrations also mostly increased especially 24 month after the after clear-cut (Tables 1 and 2); however, to a lower proportion. Typically more than 95% of the soil N is closely associated to SOM [59] and can be stabilized against decomposition by association with minerals, by its inherent recalcitrance, and by occlusion in aggregates. The soil N concentration is expected to be largely affected by clear-cutting due to several partly contrasting effects operating on different time scales. On the long-term, the removal of tree biomass in form of stems, branches, and leafs and the associated decreased litter fall and discontinuation of a steady input of N-containing biomass changes N fluxes, which can reduce total N contents in the soil [5,60]. Depending on the further development of understorey vegetation, this loss of N input into the soil can partly be diminished by the reduced N plant uptake after clear-cut. Input and incorporation of organic N via cutting debris such as needles, leafs, and branches can result in a short-time increase in organic N in the SOM. However, increased soil temperatures following clear-cutting can accelerate mineralization and nitrification and thus nutrient release from decomposing cutting debris. This can lead to increased N losses via dissolved inorganic and organic N [18,61–63] and particulate N until N fluxes are stabilized. Additional N input via slash residues however might also mask a possible boost in organic N mineralization due to increased breakdown of macroaggregates after clear-cut and the resulting higher availability and accessibility of former enclosed organic N, which is also reflected in the C:N ratio.

The generally wider C:N ratio for all sampling dates (Table 1) in the topsoils than in the subsoils might be explained by the wide C:N ratio (~40) characteristic for the biomass inputs through the Norway Spruce [62,64] being mainly incorporated in the topsoil. The observed constant wide C:N ratios in the bulk topsoil suggest that decomposability of SOM seams not affected by the clear-cut. The significant wider C:N ratio and higher total C and N concentrations in bulk subsoil 24 month after clear-cut than before clear-cut suggest a slow and delayed downward transport and accumulation of SOM into the subsoil in particulate and dissolved forms origination from the decomposition of logging and cutting debris. It is known that microbial biomass foster aggregate stability [9], hence an aggregate becomes more stable when portions of microbial biomass increases as indicated by the decrease in the C:N ratio of associated SOM. Therefore, the wider C:N ratio 24 month after clear-cut in the microaggregate size fraction of the top and subsoil than before clear-cut hint at a slow decomposition of SOM and thus less microbial contribution within microaggregate associated SOM.

To reveal clear-cut induced changes in the molecular-chemical composition of SOM in the microaggregate size fraction of the topsoil, Py-FIMS spectra were recorded, which were all dominated by *m/z* signals 392 and 436. The *m/z* 436, tentatively assigned to C_30_H_44_O_2_, monomeric syringyl units, is a characteristic marker signal of beech roots [65] and thus part of the fingerprint of the native beech tree vegetation on this stand [34]. The relative intensity of *m/z* 436 was only slightly reduced after clear-cut and remained then constant. This was different for *m/z* 392 showing the highest relative intensity 24 month after the clear-cut. The *m/z* 392 can be tentatively assigned to unsaturated ethylcholestanes being a dehydration product of phytosterols during pyrolysis, which are abundant in non-humified plant remains [66]. In addition, *m/z* 406 (long-chained lipids, octacosanol), which appeared and slightly increased with time after the clear-cut, is a typical plant-derived marker signal of the coniferous epicuticular waxes [67,68] (Fig 1). This implies that the increased input of coniferous logging and cutting residues over time led to a relative enrichment of non-degraded plant derived SOM in the microaggregate size fraction of the topsoil. This was also confirmed by the significant increase in the proportions and total intensities in the compound classes of LDIM, LIPID, and STEROL (Table 3) all assigned to be main components of spruce-tree parts [68]. The time-delayed increase in STEROL and *m/z* 392 hint to a slow break-down, transport, and incorporation of tree-cut residues like branches and leafs into the microaggregate size fraction of the mineral topsoil layer. The significant decrease in relative proportions of the compound class FATTY hint at a decarboxylation of fatty acids as was also observed in literature after clear-felling [69,70] also explaining the concomitant increase in the relative proportion and absolute intensity of the compound class LIPIDS also incorporating n-alkanes and the increase in bulk topsoil pH. The observed increase of relative proportions but also total intensities of carbohydrates after clear-cut might be explained by the additional input of the plant-derived carbohydrates from cutting residues and increased microbial biomass. To estimate the microbial contribution on SOM in the microaggregate size fraction the hexose to pentose ratio was used. The carbohydrates in the microaggregate size fraction were mainly microbial derived due to a hexose:pentose ratio >3 [46]. The significant decline of the hexose:pentose ratio in the course of clear-cutting suggest an incorporation of plant material into the soil due to slash-residues and thus an increased contribution of plant derived carbohydrates, going along with increased C:N ratios (Table 2). The significant increase of the hexose:pentose ratio and reduced proportions and absolute intensities of CHYDR 24 month after clear-cut is probably due to a continued microbial decomposing the fresh input of plant debris and labile C of new exposed aggregate surfaces released due to break-down of larger aggregates. All this is leading to an increased contribution of microbial carbohydrates with longer times after clear-cut. A higher contribution of microbial derived carbohydrates in turn tended to increase the general thermal stability of the SOM again (Fig 3) as also described in literature [46].

The often bimodal shape of the thermograms of soil samples is well described in literature [71] and reflects groups of SOM that bonds with different thermostability. Undecomposed free plant fragments and thermally labile fractions as, e.g., lignin monomers, phenols, carbohydrates, or N-containing compounds being associated with humified SOM by weak organic-mineral or organic-organic bonds, have a volatilization maximum around 350 to 380°C [72]. The thermostable compounds with a volatilization maximum ≥410°C represent the SOM fraction with strong organic-mineral or organic-organic bonds [73]. The reduced proportions of thermolabile CHDRY and NCOMP after clear-cut (Fig 2) goes along with a general deterioration of the soil aggregate structure after the clear-cut (Table 2) releasing fresh aggregate surfaces exposing labile SOM originally protected against turnover [9]. The increasing proportion of thermolabile compound classes such as STEROL and reduction of thermostable proportions as observed for LIPID after clear-cut is an effect of the introduction of plant constituents into the soil following the event of clear-cutting [72]. The minor effect of clear-cut on volatilization curves of PHLM, LDIM, and PEPTI suggests that not all compounds of SOM are equally influenced by the change in environment, with the stability of more labile OM compound classes more sensitive to land-use changes.

### 4.2 Influence of the clear-cut on P and S availability and binding forms

Generally, the P concentrations in the microaggregate size fraction of different top- and subsoil P pools were in a comparable range as described in literature for mineral bulk top- and subsoils of temperate forest soils [74]. Higher proportions of P_HCl_ in the subsoil than in the topsoil hint at a higher contribution of P from primary P minerals such as Ca-P minerals. However, due to the low pH values other P sources such as Mg-P minerals, hydrolyzed Po during extraction, and/or occluded P are also likely to contribute to this pool. Furthermore, the dominance of the NaOH-extractable P and the higher contribution of Po than Pi in P_HCO3_ and P_OH_ pools (Table 4) suggest a stabilization of Po (i) in the microbial biomass and (ii) via adsorption to soil Fe and/or Al minerals. This hints to a slow resupply of P from mineral weathering [75] but and effective Po recycling [76].

The dominance of Fe- and Al-associated P as revealed by XANES spectroscopy (Table 5) is in agreement to literature showing that in acidic mineral forest soils layers on silicate parent materials main P forms are Fe- and Al bound P species [77]. High proportions of Fe- and Al-associated P (between 68 to 81%, Table 5) were not reflected in P proportions of Pi_HCO3_ + Pi_OH_ (ranging between 16 to 32%, Table 4) representing P associated to pedogenic Al- and Fe-(hydr)oxides [29,30]. This overestimation of inorganic Fe- and Al- associated P by XANES spectroscopy originates from the difficulty to differentiate whether Pi or Po is sorbed to Fe with this method due to the fact that both Pi and Po sorbed or chemically bound to Fe lead to the known pre-edge peak in the XANES spectrum and therefore cannot clearly be distinguished in the LC-fitting of the spectra [31]. Therefore, it is very likely that proportions of P sorbed to Fe determined by P XANES also include Po sorbed to Fe also explaining the underestimation of Po by XANES spectroscopy compared to sequential fractionation results, especially for Po rich samples.

The high contribution of P sorbed to ferrihydrite rather than to goethite can be explained by the high OC contents of the microaggregate size fraction. It is known that high OC contents (>5%) inhibits crystallization of short-ranged ordered Fe to more crystalline forms such as goethite and hematite [78] due to the sorption of organic anions and thus prevention of nucleation [79]. Subsoil proportions of P sorbed to gibbsite and ferrihydrite were similar to proportions of the topsoil microaggregate size fraction (Table 6), being in agreement to insignificant differences of NaOH extractable P concentrations revealed by sequential fractionation (Table 4). This is in agreement to literature stating comparable or slightly lower proportions of P associated to Al- and Fe-(hydr)oxides between the A and B horizon of comparable acidic Cambisol soils under forest [77].

In literature it is stated that aggregate break-down due to clear-cutting led to an increased turnover of associated SOM and thus nutrients stored therein [68]. Especially P dynamics are influenced by the removal of trees [19]. Due to the break-down of macroaggregates exposed P becomes accessible to microbes and plants [10]. In the short-term, biological turnover mostly occurs from labile P bonding forms as represented by the P_Resin_ and the P_HCO3_ pool. Sequential fractionation of the top- and subsoil microaggregate size fraction revealed a decreasing trend in proportions and concentrations of P_Resin_ after clear-cut, which however were not significant. This reduced directly available P pools despite increasing total P concentrations is probably a results of an increased microbial activity as indicated by the hexose to pentose ratio after clear-cut and thus increased P turnover [10] and immobilization within microbial biomass as also reflected in the hexose:pentose ratio obtained by Py-FIMS analyses.

No clear effects of clear-cutting were visible in proportions of different P forms obtained by XANES spectroscopy. An exception to this is an enrichment of Po in the topsoil 10 month after clear-cut, most likely due to the introduction of organic clear-cut debris. However, this was not reflected in the corresponding total Po data obtained from the sequential P fractionation. This can partly be explained by the higher proportion of residual-P 10 month after clear-cut, which is thought to be mainly Po and is included in Po proportions detected by XANES spectroscopy but not in the total Po data obtained from the sequential P fractionation.

In addition, when calculating the C and Po ratio (C:Po) we observed an increase of the ratio in the topsoil microaggregate size fraction samples from 328 (Control), to 494 (month 10) and 441 (month 24), whereas in the subsoil the ratio was 68 (control), 35 (month 10), increasing to 590 (month 24). The C:Po ratio is an indicator for the dominant processes influencing the distribution of Pi and Po in soils, with low C:Po ratios (<200) hinting at a mineralization of Po and thus a release of available Pi, whereas high ratios (>300) are indicative for an immobilization of P by incorporation into biomass [80,81]. Topsoil microaggregate C:Po ratios >400 found after the clear-cut thus indicate a shift towards more stable P-pools and an immobilization within microbial biomass, which is in agreement to Py-FIMS results indicating a higher microbial contribution to SOM after clear-cut. The lower C:Po ratio in the subsoil than in the topsoil can be attributed to the sharp decrease of organic C compared to P. However, 24 month after the clear-cut organic C concentrations and thus the ratio C:Po increased to >500 in the subsoil and thus also exceeding topsoil ratios. This might originate from a time-delayed incorporation of dissolved organic compounds from the slash residues into the subsoil microaggregate size fraction.

The total S concentration in the bulk soil samples (370 to 510 mg kg^-1^) was in in a comparable range of total S found in literature for other temperate forest soil [82–84]. The used extractions scheme was capable to extract in mean 79% of the total S, while around 21% was residual-S. Generally, the extraction scheme is suitable to determine the availability of S to plants [27,28], whereas it does not allow a differentiation between inorganic and organic S forms and to separate fractions into C-bonded and esters-S, as being possible when applying the traditional HI reduction method [85]. However, most S in topsoils is present as organically bound S (up to 95% of total S) as reviewed by [86] and references therein, whereas subsoil S is mainly composed of mineral S compounds [87], which is also assumed to be the case of the here studied subsoil. However, limitations of organic and inorganic S differentiation are partly overcome by complimentary application of S *K*-edge XANES spectroscopy to further speciate different binding forms of S [53].

Most of the directly plant-available inorganic SO_4_^2-^ fraction being in soil solution is extracted within the S_Resin_ pool. Proportions of S_Resin_ determined by sequential S fractionation (Table 5) were lower than proportions of sulfate (including organic and inorganic sulfates) estimated by XANES spectroscopy (Table 7). A direct comparison between plant available S fractions determined by sequential fractionation (S_Resin_, S_HCO3_) to specific S species determined by XANES spectroscopy is not possible; however, water-soluble sulfates are directly available to plants, while the mineralization of sulfate from organic sulfur, especially from ester sulfate, also makes an important contribution to the plant available S fraction [88]. Thus, it is assumed that a substantial part of short-term to intermediate plant available S is quantified within the sulfate proportions, being also the main fraction of S identified by S XANES spectroscopy. The higher SOM concentration in the topsoil was also reflected by higher diversity of S forms also including substantial proportions of reduced S forms (disulfides, thiol, thiophenes), which were absent in the subsoil. In the subsoil the sulfate S almost is the dominating (91 to 100%) S form (Table 7), being in accordance to literature that subsoil S mainly constitutes mineral S [87]. The coprecipitation with calcium as insoluble inorganic sulfates can be neglected due to the low concentrations of Ca due to the inherent lack of carbonates in acidic mineral forest soil layers as was also confirmed by S XANES spectroscopy, as characteristic post-edge features of Ca-sulfates [89] were not observed in the spectra.

The significant increase in total S in the microaggregate size fraction of the topsoil 24 month after the clear-cut (Table 2) hints at an additional input of S by leaching from cutting residues combined with a reduced S uptake by trees after clear-cut. This delayed increase in total S results from the delayed incorporation of leachates of cutting residues into the mineral soil horizons as residues must be first mechanically or microbially decomposed in the overlaying organic horizons before it can leach into the underlying mineral soil. This corresponds to the delayed significant increase of available S pools 24 month after clear-cut in the top- and subsoil (Table 5), which is also amplified by the reduced plant-uptake after tree-removal and secondly by the deteriorated soil structure visible in the increase of the soil microaggregate size fraction (Table 2). As already described above for P the break-down of larger aggregates can also have led to a release of originally inaccessible S to the attack by soil microorganisms and thus increasing the accessibility and available S fraction in top- and subsoils. This also explains the concomitant reduction of the stable S pool (S_HCl_). However, there is a lack of knowledge about the binding forms of exposed S after aggregate breakdown. As stated by Solomon et al. [53] break-down of aggregates after land-use change reduced proportions of reduced S forms in soils. Therefore, the observed higher proportions of reduced S forms (especially with oxidation state S^-2^) in the topsoil 10 month after clear-cut may at least partially originate from the exposure of reduced S formally enclosed in the macroaggregates. Furthermore, decreased proportions of reduced S forms and increased proportion of intermediate S (Table 6) after 24 month suggest a progressing oxidation of the exposed reduced S forms after clear-cut.

An incorporation of organic slash residues into the mineral topsoil layer also explains the more than doubling of the proportion of sulfonate 10 month after the clear-cut (Table 7). Sulfonate is known to directly derive from leaf litter and root inputs [53]. The lack of sulfonate quantification 24 month after clear-cut can be attributed to a stopped input of leaf-litter and slash residues and a decomposition of already existing sulfonate.

## 5 Conclusions

The clear-cut led to a deterioration of the aggregate structure accompanied by increased hexose to pentose ratio suggesting and increased microbial activity leading to an increased turnover of P and S in the microaggregate size fraction. While input of slash residues generally led to an increase of SOM, this SOM was of lower thermal stability and thus did not contribute to general aggregate stability of the system. However, break-down of aggregates led to enhanced availability of organic C, N, P, and S. This caused an increased probably, releasing microbial derived carbohydrates to the soils, which in turn increased aggregate stability. This indicated that although clear-cutting first led to a break-down of aggregates, with time a recreation of the aggregate structure and thus formation of even more stable aggregates can occur. However, changes of aggregate structure and P and S speciation with increasing time after clear-cut are mostly significant but rather small leading to the assumption that short-term effects do not have to be considered as too drastic as expected for this specific site. Nevertheless, long-term effects cannot be deduced from these results and thus further long-term monitor studies are required of the soil structure development in order to see whether these are only short-term effects on the way to a new equilibrium.
